# Achievement of Cardiometabolic Goals among Diabetic Patients in Spain. A Nationwide Population-Based Study

**DOI:** 10.1371/journal.pone.0061549

**Published:** 2013-04-18

**Authors:** Beatriz Navarro-Vidal, José R. Banegas, Luz M. León-Muñoz, Fernando Rodríguez-Artalejo, Auxiliadora Graciani

**Affiliations:** Department of Preventive Medicine and Public Health, Universidad Autónoma de Madrid/IdiPAZ; CIBERESP, Madrid, Spain; University of Chieti, Italy

## Abstract

**Background:**

No previous study has reported a comprehensive assessment of the attainment of cardiometabolic goals in the diabetic population of a European country. We examined the achievement of cardiometabolic goals among diabetics in Spain.

**Methods and Findings:**

A cross-sectional survey was performed in 2008–2010 among 12,077 individuals representative of the Spanish population aged ≥18 years. Information on cardiometabolic characteristics was collected at the participants’ homes through structured questionnaires, physical examination, and fasting blood samples. Attainment of cardiometabolic goals was evaluated according to the most well-known guidelines. A total of 834 individuals had diabetes (fasting serum glucose ≥126 mg/dl, or glycosylated hemoglobin ≥6.5%,) or were being treated with oral antidiabetic drugs or insulin). Among diabetic patients, 661 (79.2%) were aware of their condition. Among the aware diabetic patients, only 11.4% had neither general (body mass index <25 kg/m^2^) nor abdominal obesity (waist circumference ≤102 cm in men and ≤88 cm in women), 8.6% consumed <7% of calories daily from saturated fats, and 41.1% achieved the recommendation on weekly physical activity. About 71% had glycosylated hemoglobin <7%, 22% had blood pressure <130/80 mmHg, and 36% reached the LDL-cholesterol goal of <100 mg/dl. Although a large proportion of aware diabetic individuals received lifestyle medical advice, only 38% of overweight individuals and 20% of daily smokers were offered a specific strategy for weight loss or quitting smoking, respectively.

**Conclusions:**

In a European country with universal healthcare coverage, achievement of many cardiometabolic goals, in particular lifestyle, among aware diabetic individuals is poor. This suggests a need for improvement in both clinical guidelines' implementation and patients’ adherence.

## Introduction

The control of cardiovascular risk factors, either through lifestyle or drug treatment, have shown health benefits for diabetic patients and those at high risk of diabetes [1]. Thus, American and European clinical practice guidelines on diabetes management have focused not only on glycemic control, but also on modification of lifestyles and achievement of cardiovascular goals in diabetic patients [2]–[4].

Most previous studies have focused on the control of a few cardiovascular risk factors in persons with diabetes, generally, serum glucose, blood pressure (BP), and lipids [5]–[9]. Moreover, only some of these studies were population-based and representative of an entire country [5]–[7].

Thus, our objective was to examine the achievement of main cardiometabolic goals among diabetic subjects in a representative sample of the adult population of Spain. These goals include lifestyles (not smoking, being physically active, having normal body weight, reduced intake of dietary fat), biological variables (control of blood glucose, blood pressure, LDL- cholesterol and albumin excretion) and provision of health services (medical advice on lifestyle, and treatment with angiotensin converting enzyme (ACE) inhibitors, angiotensin II receptor blockers (ARB), statins or aspirin, if indicated) [2]–[4]. To our knowledge, no published study has reported a comprehensive assessment of the attainment of cardiometabolic goals in the diabetic population of a European country.

## Methods

### Ethics Statement

The study protocol was approved by the Clinical Research Ethics Committee of the University Hospital *La Paz* in Madrid and the Hospital *Clínic* in Barcelona. All the patients provided written informed consent for the study.

### Research Design and Methods

The data were taken from the Study on Nutrition and Cardiovascular Risk in Spain, whose methods have been reported elsewhere [10]. In brief, this was a cross-sectional study conducted from June 2008 through October 2010 on 12,948 individuals representative of the non-institutionalized population of Spain aged ≥18 years.

Study participants were selected by multistage clustered random sampling. The sample was first stratified by province and by municipality size. Clusters were then selected randomly in two stages: municipalities and census sections. Finally, the households within each section were selected by random telephone dialing (landline telephone directory as sampling frame). Subjects in the households were selected proportionally to Spanish population distribution by sex and age. Information was collected by telephone interview, and through face-to-face interview, physical examination, and collection of blood and urine samples in the households.

Diabetes was defined as 12-hour fasting serum glucose ≥126 mg/dl or glycosylated hemoglobin (HbA1c) ≥6.5%, or being treated with oral antidiabetic drugs or insulin [2]. Among diabetic individuals, awareness of their condition was defined as a positive response to the question: “Have you ever been told by the doctor that you had diabetes or elevated blood sugar?” Glycemic control was defined as glycosylated hemoglobin (HbA_1c_) <7% [2]. Treatment was defined as reported current use of diabetes drug therapy.

Study participants reported their smoking status. Physical activity and habitual food consumption were based on questionnaires validated in the EPIC cohort of Spain [10]–[12]. The nutrient intake was estimated using composition tables for Spanish and foreign foods [10], [13], [14]. Individuals also reported physician-diagnosed morbidity, in particular, ischemic heart disease, stroke or heart failure, and the use of health care.

Weight, height, and waist circumference (WC) were measured twice in each subject under standardized conditions [15], using electronic scales (model Seca 841, precision to 0.1 kg), portable extendable stadiometers (model Ka We 44 444Seca) and flexible, inelastic belt-type tapes. Body mass index (BMI) was calculated as weight in kg divided by squared height in m. BP was measured, using standardized procedures [16], with validated automatic devices (model Omron M6) and cuffs of three sizes according to arm circumference. Two sets of BP readings were made separated by 90 minutes. In each set, BP was measured three times at 1–2 minute intervals. In the analyses, BP was calculated as the mean of at least three out of the last five readings. Hypertension in the known diabetic population was defined as systolic BP (SBP) ≥130 mm Hg, diastolic BP (DBP) ≥80 mm Hg, or on current antihypertensive medication.

LDL cholesterol was calculated by the Friedewald formula, where total cholesterol was measured by the cholesterol-esterase and cholesterol-oxidase method; HDL cholesterol by the direct method, by elimination/catalase; and triglycerides by the glycerol phosphate oxidase method. Creatinine was determined by the Jaffé method (rate-blanked), kinetic reaction. Microalbumin was determined by PEG enhanced immunoturbidimetry. Glucose was measured by the glucose oxidase method. The analyzer ADVIA 2400 Chemistry System from Siemens was used for all the above parameters. HbA_1c_ was measured by chromatography by HPLC (analyzer ADAMS A1c HA-8160, Arkray). The laboratory tests were performed centrally at the Center of Biological Diagnosis of the *Hospital Clínic* in Barcelona, according to standard procedures and incorporating appropriate quality controls.

Study participants were asked if they had ever been advised by a physician on lifestyles (diet, salt reduction, physical activity, weight loss, quitting smoking), with three possible responses: 1) No; 2) Yes, but I do not follow it; and 3) Yes, and I do follow it. A positive response to 2 or 3 was considered as receiving advice, and a positive response to 3 was considered adherence to the advice. A nurse also recorded each participant’s medication use.

### Cardiometabolic Goals

We classified the cardiovascular health status and management of diabetic patients into five categories: lifestyle, biological factors, medical advice on lifestyles, cardioprotective treatment, and use of health care; and goals were taken from well-known guidelines in effect at the time of the survey [2]–[4], [17]. Among unaware diabetic subjects, the goals were those recommended for the general population [3], [17].

Lifestyle goals were set as: non-smoker (never or ex-smoker); normal weight (BMI <25 kg/m^2^) and lack of central obesity (WC ≤102 cm in men and ≤88 in women); moderate or vigorous physical activity (≥150 minutes/week of moderate intensity or ≥60 minutes/week of vigorous intensity); and reduced energy intake from saturated fat (<7% in aware diabetes and <10% in unaware diabetes).

Biological goals were defined as control of: blood glucose (HbA_1c_ <7%); systolic and diastolic blood pressure (<130/80 mmHg in aware diabetes and <140/90 in unaware diabetes); LDL cholesterol (<100 mg/dl in aware diabetes and <115 mg/dl in unaware diabetes); and albumin excretion (urinary albumin:creatinine ratio <30 mg/g).

Medical advice goals were receiving advice on and being adherent to: diet to control blood glucose in aware diabetics and salt reduction in known hypertensives, weight reduction in subjects with overweight (BMI ≥25 kg/m^2^), at least moderate physical activity, and smoking cessation.

Cardioprotective treatment goals were being treated with: ACE inhibitors/ARBs in known hypertensives; statins, if indicated (i) overt cardiovascular disease (CVD); ii) no CVD and age over 40 with one or more other CVD risk factors; iii) no overt CVD and age under 40 if LDL cholesterol remains >100 mg/dl, or multiple CVD risk factors); aspirin <500 mg/day, if indicated (history of CVD and age over 40 or additional risk factors, including family history of CVD, hypertension, smoking, dyslipidemia, or albuminuria); and annual influenza immunization (in all aware diabetics and in unaware diabetics aged 60 years and older).

Lastly, a goal on health care use was set as glucose testing ≥2 times per year.

### Statistical Analysis

From the 12,948 study participants, 871 were excluded due to lack of valid information on the variables of interest. Thus, the analyses on the prevalence of diabetes were conducted with 12,077 individuals. To determine the achievement of cardiometabolic goals according to diabetes awareness status, we limited the analyses to the 661 aware diabetic patients and 173 unaware diabetic patients with complete information on variables of interest.

The analyses took account of the complex sampling design; thus, individual observations were weighted to reconstruct the Spanish population, and the variances were corrected to obtain appropriate 95% confidence intervals (CI) for the main results. Categorical variables were reported as a percentage and continuous variables as a mean, except for albumin excretion which was summarized with the median. The *X* ^2^ test was used to compare percentages; the Student *t* test to compare means; and the Mann-Whitney *U* test to compare medians. The associations between each cardiovascular health goal (yes/no) as outcome and awareness of diabetes (unawareness as reference) as primary covariate, were summarized with odds ratios (OR) and their 95% confidence interval (CI), obtained from logistic regression. Analyses were adjusted for age (continuous), gender, and educational level (less than high school, high school and university).

Statistical significance was set at two-sided p<0.05. The analyses were performed with the *survey* procedure in STATA®, version 11.2.

## Results

Among the 12,077 participants, 834 (6.9%) had diabetes, of whom 661 (79.2%) were aware of their condition. Mean age of the aware diabetic subjects was 64.4 years, 41.7% were women, and 57.7% had less than high school. Mean diabetes duration was 7.7 years and mean HbA_1c_ was 6.5% (Table 1). Unaware and aware diabetics had similar demographic characteristics in terms of gender and educational level. However, unaware diabetics were somewhat younger that aware diabetic individuals (Table 1). As regards clinical characteristics, both groups had comparable figures, except for HbA_1c_ and urinary albumin excretion which were lower in unaware diabetics, and for total and LDL cholesterol which were lower in aware diabetics (Table 1). Eighty five percent of aware diabetics were non-smokers (Table 2). Only 13.2% had normal weight (BMI<25 kg/m^2^) and less than one-third did not have abdominal obesity. As a result, 11.4% had neither general nor abdominal obesity. About 8.6% consumed less than 7% of calories from saturated fat, and 41.1% met the recommendations for moderate or vigorous physical activity (Table 2). Glycemic control (HbA_1c_<7%) was achieved in 70.9% of aware diabetic patients vs. 84.3% of unaware diabetics (p = 0.002). Only 21.9% of aware diabetics met the BP target (<130/80 mmHg) compared with 29.2% of unaware diabetics (p = 0.086). Seven out of ten aware diabetics and eight out of ten unaware diabetics had a normal urinary albumin excretion (urinary albumin:creatinine ratio <30 mg/g) (p = 0.028). In contrast, 35.6% of aware diabetics reached the LDL cholesterol goal of <100 mg/dl, while only 29% of unaware diabetics met the goal for the general population (LDL-cholesterol <115 mg/dl) (Table 2).

**Table 1:** Demographic and clinical characteristics among aware and unaware diabetic individuals.

	Aware diabetes n = 661	Unaware diabetesn = 173	*p*
**Sex (%)**			
Women	41.7	42.2	0.920
Age (years)	64.4	62.2	0.151
**Age (%)**			
18–44	7.2	16.2	0.004
45–64	37.0	30.3	0.149
≥65	55.7	53.4	0.651
**Education (%)**			
Less than high school	57.7	51.7	0.230
High school	24.9	31.9	0.110
University	17.4	16.3	0.771
**Tobacco smoking in daily smokers**			
(No. of cigarettes per day)	15.6	19.5	0.138
**Body mass index (kg/m^2^)**	30.2	30.4	0.666
**Waist circumference (cm)**			
Men	105.8	104.3	0.297
Women	99.1	98.5	0.733
**Physical activity** a **(min/week)**	139.2	154.7	0.432
**Calorie intake from saturated fat (kcal)**	234.4	248.6	0.302
**Fasting glucose (mg/dl)**	143.1	142.8	0.926
**HbA_1c_ (%)**	6.5	6.0	<0.001
**Duration of diabetes (years)**	7.7	–	
**Blood pressure (mmHg)**			
Systolic	142.6	140.1	0.227
Diastolic	77.4	78.8	0.218
**LDL Cholesterol (mg/dl)**	112.6	126.7	<0.001
**Urinary albumin:creatinine ratio (mg/g)**	8.0	6.0	0.028

Data are presented as means for continuous variables and percentages for categorical variables, except for Urinary albumin:creatinine ratio presented as median.

a Moderate or vigorous physical activity: ≥150 minutes/week of moderate intensity or ≥60 minutes/week of vigorous intensity.

*p-values* were calculated by a *X^2^* test to compare percentages, a Student *t* test to compare means and a Mann-Whitney test to compare medians.

**Table 2:** Cardiometabolic health status among aware and unaware diabetic individuals.

	Aware (n = 661)		Unaware (n = 173)		
	%	95% CI	%	95% CI	*p*
**Lifestyles factors**					
Non-smokers	84.6	81.6–87.7	80.5	74.0–87.0	0.234
Body mass index <25 kg/m^2^	13.2	10.3–16.2	12.7	6.7–18.7	0.882
Body mass index <30 kg/m^2^	53.3	48.8–57.8	50.0	41.1–58.9	0.505
Waist circumference ≤102/≤88 cm (men/women)	29.4	25.4–33.5	31.3	23.0–39.6	0.691
BMI <25 kg/m^2^ and waist circumference ≤102/88 cm	11.4	8.6–14.2	12.7	6.7–18.8	0.688
Physical activity (moderate or intense^a^)	41.1	36.5–45.7	45.8	37.4–54.2	0.333
Calorie intake from saturated fat <7%	8.6	6.2–11.1	5.8	2.2–9.4	0.245
Calorie intake from saturated fat <10%	37.7	33.4–42.0	37.0	28.5–45.5	0.875
**Biological factors**					
HbA_1c_<7%	70.9	66.8–75.0	84.3	78.1–90.6	0.002
Blood pressure <130/80 mmHg	21.9	18.1–25.6	29.2	21.1–37.4	0.086
Blood pressure 130–139/80–89 mmHg	28.1	23.9–32.5	24.5	17.1–31.9	0.411
LDL cholesterol <100 mg/dl	35.6	31.3–39.9	22.1	14.4–29.8	0.006
LDL cholesterol <115 mg/dl	53.7	49.0–58.4	29.1	21.0–37.1	<0.001
Urinary albumin: creatinine ratio <30 mg/g	76.4	72.5–80.3	85.7	79.5–91.8	0.028
**Medical advice**					
Diet to control diabetes	86.7	83.6–89.8	–	–	–
Adherence to diet to control diabetes	75.1	71.2–78.9	–	–	–
Reduction of salt consumption in known hypertensives	88.7	85.1–92.3	80.7	70.8–90.6	0.089
Adherence to reduction of salt consumption in known					
hypertensives	81.9	77.5–86.4	74.8	63.8–85.8	0.209
Weight reduction (if BMI ≥25 kg/m^2^)	74.5	70.2–78.8	62.9	54.1–71.7	0.013
Adherence to weight reduction (if BMI ≥25 kg/m^2^)	45.5	40.5–50.4	32.5	23.3–41.6	0.019
Weight loss strategy	38.3	33.6–42.9	27.6	19.4–35.8	0.035
Physical activity at least moderate	85.2	81.6–88.9	70.5	62.4–78.6	<0.001
Adherence to physical activity at least moderate	64.5	59.8–69.2	43.3	34.8–51.9	<0.001
Stopping smoking (if daily smokers)	86.4	78.6–94.1	64.5	44.7–84.4	0.021
Smoking cessation strategy (if daily smokers)	19.6	9.2–30.0	8.5	3.1–20.1	0.230
**Cardioprotective treatment**					
Only oral antidiabetics	64.4	60.0–68.7	–	–	–
Only insulin	10.8	7.9–13.7	–	–	–
Oral antidiabetics and insulin	9.3	6.8–11.8	–	–	–
Treatment with ACE inhibitors/ARBs in known					
hypertensives	58.8	53.0–64.6	54.8	41.4–68.2	0.580
Treatment with statins (if indicated^b^)	37.7	33.2–42.3	28.7	19.9–37.6	0.086
Treatment with aspirin <500 mg/day (if indicated^c^)	15.3	12.1–18.6	6.8	1.7–11.9	0.028
Annual influenza immunization	60.1	55.5–64.8	46.2	37.1–55.3	0.007
Annual influenza immunization in persons >60 years	68.9	63.7–74.0	60.9	50.2–71.6	0.176
**Use of health services**					
Glucose testing 2 or more times per year	53.9	49.2–58.6	38.5	29.7–47.4	0.003

a Moderate or vigorous physical activity: ≥150 minutes/week of moderate intensity or ≥60 minutes/week of vigorous intensity.

b Statin indications: i) Overt cardiovascular disease (CVD), ii) no CVD and age over 40 with one or more other CVD risk factors, iii) no overt CVD and age under 40 if LDL cholesterol remains >100 mg/dl, or multiple CVD risk factors.

c Low-dose aspirin indications: history of CVD and age over 40 or additional risk factors (family history of CVD, hypertension, smoking, dyslipidemia, or albuminuria).

*p-value* was calculated by a *X^2^* test to compare percentages.

A large percentage of study participants received medical advice on lifestyles, although adherence to such advice was low (Table 2). Only 38.3% of overweight individuals and 19.6% of daily smokers, who knew their diabetes status, were offered a specific strategy for weight loss or quitting smoking, respectively. For those unaware of their condition, these specific strategies were recommended even less frequently (Table 2).

About 59% of aware diabetic individuals with known hypertension were being treated with ACE inhibitors or ARBs. Only 37.7% of those with statin indication were treated with these drugs, and as few as 15.3% of those with a clear indication were being treated with low-dose aspirin. Compared with aware diabetics, those unaware of their condition were significantly less likely to be treated with statins or aspirin (Table 2). Around 69% of persons over age 60 with aware diabetes and 61% with unaware diabetes had been vaccinated against influenza in the previous year. Lastly, over one third of unaware diabetics had their blood glucose levels tested more than twice a year, in contrast with only half of aware diabetic subjects (p = 0.003) (Table 2).

Table 3 shows the adjusted association between diabetes awareness and achievement of selected cardiometabolic goals. Unaware diabetic patients showed a better glycemic control and renal function, while aware diabetic subjects more frequently had LDL cholesterol controlled, adhered to advice on weight reduction and physical activity, and received statin and aspirin treatment.

**Table 3:** Achievement of selected cardiometabolic goals among aware vs. unaware diabetic individuals.

		95%	
	Odds ratio^a^	confidence interval	p
**Lifestyles**			
Non-smokers	1.16	0.73–1.84	0.537
BMI<25 kg/m^2^ and waist circumference ≤102/88 cm	0.98	0.58–1.65	0.933
Moderate or intense physical activity	0.88	0.62–1.25	0.479
Calorie intake from saturated fat <7%	1.49	0.73–3.02	0.271
Calorie intake from saturated fat <10%	0.97	0.68–1.39	0.884
**Biological factors**			
HbA_1c_<7%	0.41	0.26–0.65	<0.001
Blood pressure <130/80 mmHg	1.31	0.87–1.96	0.196
Blood pressure <140/90 mmHg	0.95	0.67–1.35	0.771
LDL cholesterol <100 mg/dl	1.93	1.29–2.90	0.001
LDL cholesterol <115 mg/dl	2.81	1.94–4.08	<0.001
Urinary albumin:creatinine ratio <30 mg/g	0.56	0.35–0.89	0.016
**Medical advice**			
Reduction of salt consumption in known hypertensives	1.88	0.96–3.65	0.064
Adherence to reduction of salt consumption in known hypertensives	1.48	0.82–2.67	0.192
Weight reduction (if BMI ≥25 kg/m^2^)	1.81	1.23–2.68	0.003
Adherence to weight reduction (if BMI ≥25 kg/m^2^)	1.84	1.25–2.72	0.002
Weight loss strategy	1.77	1.18–2.66	0.006
Physical activity at least moderate	2.42	1.63–3.60	<0.001
Adherence to physical activity at least moderate	2.36	1.67–3.33	<0.001
Advice to stop smoking in daily smokers	3.89	0.51–29.8	0.191
**Cardioprotective treatment**			
Treatment with ACE inhibitors/ARBs in known hypertensives	1.22	0.74–2.02	0.443
Treatment with statins if indicated	1.47	1.01–2.15	0.048
Treatment with aspirin <500 mg/day if indicated	2.50	1.27–4.89	0.008
Annual influenza immunization in persons >60 years	1.44	0.93–2.22	0.100
**Use of health services**			
Glucose testing 2 or more times per year	1.86	1.31–2.63	<0.001

BMI indicates body mass index; ACE, angiotensin convertin enzime; ARB, angiotensin II receptor blocker.

a Odds ratios among aware diabetic patients vs. those unaware (reference) were adjusted for age, sex, and educational level.

**Figure 1:**
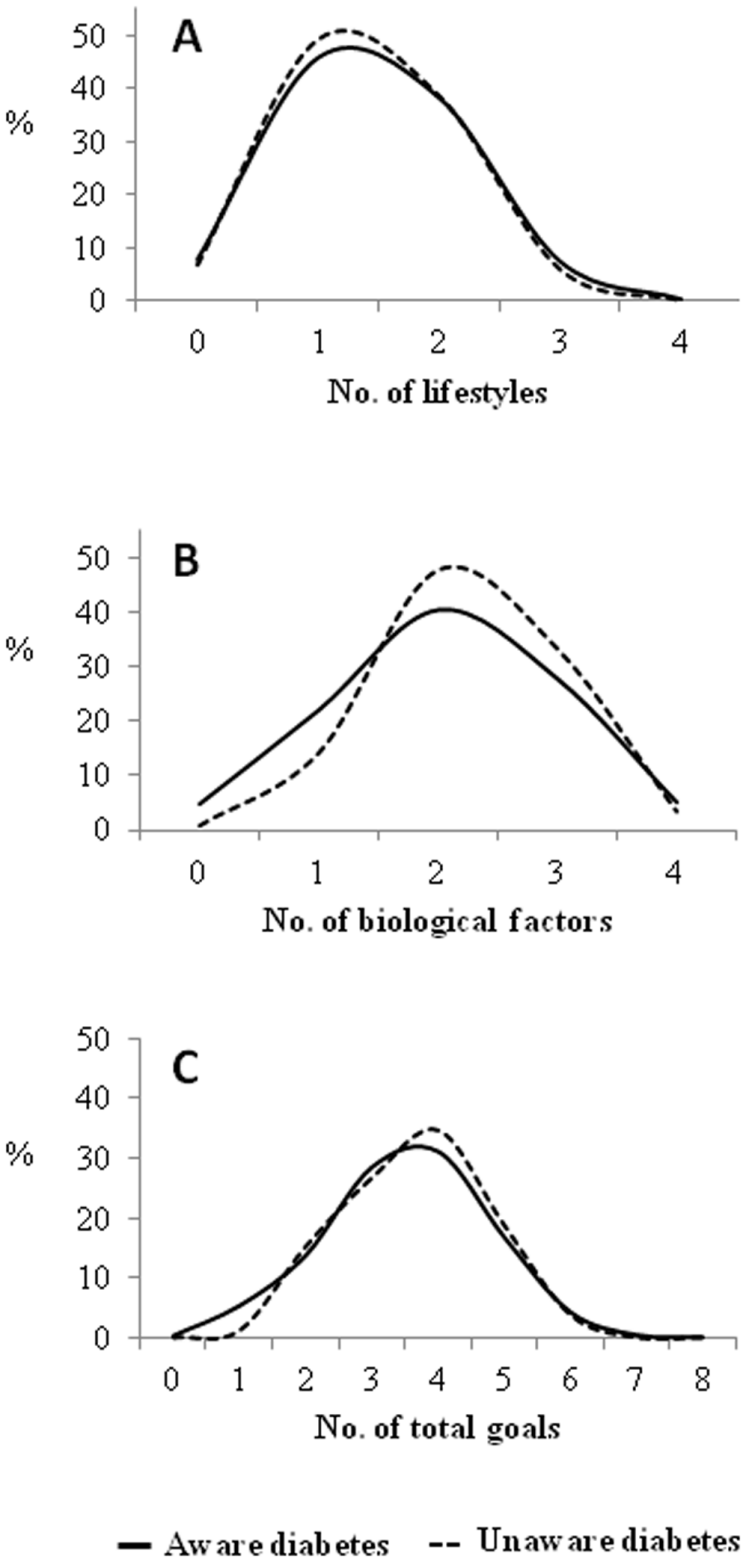
Percent distribution of the number of cardiometabolic goals (lifestyles and biological factors) met among diabetic patients. **A.-** Lifestyle: non-smoker; BMI <25 kg/m^2^ and waist circumference ≤102/88 cm in men and women, respectively; moderate or intense physical activity; energy intake from saturated fat <7%. **B.-** Biological factors: HbA_1c_<7%; blood pressure <130/80 mmHg; LDL cholesterol <100 mg/dl; urinary albumin:creatinine ratio <30 mg/g **C.-** Lifestyle and biological factors.

Figure 1 shows the percent distribution of the number of cardiometabolic goals met among diabetic patients. About 39% of both aware and unaware diabetic subjects met two of the four lifestyle goals (Figure 1, A). As regards biological goals, 90% of the unaware diabetics reached three or more goals vs. 70% of aware diabetics (Figure 1, B). None attained all eight lifestyles and biological factor goals, and only 3 known diabetics achieved seven of them (Figure 1, C).

## Discussion

We examined the achievement of cardiometabolic goals among diabetics in Spain, and found a situation with large room for improvement: only three out of five aware diabetics met at least three of the studied goals.

Of note is the small percentage of aware diabetics who meet the American Diabetes Association (ADA) [2] recommendations on intake of saturated fats. This could be explained by a deterioration in eating habits in Spain during recent decades, which have progressively departed from the traditional Mediterranean dietary pattern [18]. In our study, as few as 9% of diabetic patients consumed <7% of calories from saturated fats, and only 6% consumed <10%. These figures are lower than those found in other studies (37.7% vs. 48.3% respectively, in the US) [5]. Besides, half of aware diabetic patients had a BMI<30 kg/m^2^, which was higher than that reported for the U.S. (37.6%) [5], but lower than those observed in several Southern European countries (over 60%) [8], [9].

However, achievement of the recommendation on weekly physical activity was higher in our study than that reported by Resnick et al. in the U.S. (41.1% vs. 28.2%) [6]; and the majority of aware diabetic patients were non-smokers, a situation similar to that in the U.S., where 82.4% of diabetics do not smoke [5].

Glycemic control in Spain is reasonably high, (71% of aware diabetic patients had HbA1C <7% vs. 55.2% in the U.S. [5]), although only one in five had their BP strictly controlled. BP control among our aware diabetic patients is far lower than in the U.S., where 40% of subjects had BP<130/80 mmHg [6]. One-third of people with aware diabetes had LDL-cholesterol <100 mg/dl, again lower than in U.S. studies [5], [19], that could be explained by less use of statins in Spain. On renal function, about 77% of persons with known diabetes had a urinary albumin:creatinine ratio of <30 mg/g as compared to 65% in the U.S. studies [5], [6], which could be due to the shorter duration of diabetes in our study (7.7 years vs. 11.4 years, respectively) and better glucose control. The large proportion of aware diabetic patients who received advice on lifestyles is similar to that in other studies in Europe [20], and the U.S. [21], [22]. Reported compliance with the advice was also quite high, except for weight reduction. However, prescription of a specific method to lose weight or stop smoking was low, especially in the latter case, which was received by only one of every six daily smokers. The weight advice data are consistent with the small percentage of individuals not achieving normal weight.

We found higher levels of treatment with oral antidiabetics than in other U.S. and European studies [5], [23]. In contrast, treatment with insulin is lower than in those countries [23]. The percentage of use of ACE inhibitors or ARBs in aware diabetic persons with known hypertension in our study was relatively low (58.8%) considering that, according to recommendations [2], [4] all such persons should receive hypertensive treatment with one drug from this group. Our data are similar to those of the U.S. [24] and much lower than the levels of use of ACE inhibitors or ARBs in hypertensive diabetics in France (82%) [25]. The use of statins in persons who require this treatment due to their cardiovascular risk or LDL levels follows the same pattern, far below the ADA recommendations and lower than found in other studies [19]. Especially low was the treatment with low-dose aspirin as an antiplatelet drug where recommended among aware diabetic patients [26], [27]. The levels of influenza immunization were moderate. Around 60% of subjects with aware diabetes were vaccinated in the previous year. In middle-aged subjects, the levels were similar to those of the U.S. [28].

In our study, being aware with diabetes was not associated with an expected better achievement of the goal for HbA_1c_ and urinary albumin:creatinine ratio. Part of the explanation could be that those unaware of their diabetes are slightly younger and have had the disease for less time (data not shown), therefore their renal function and HbA_1c_ are better preserved. Conversely, being aware of their diabetes condition was associated with more frequent control of LDL, medical advice to modify lifestyles, cardioprotective treatment and use of health services. Interestingly, after adjusting for age, gender and educational level, no differences were found between those with aware and unaware diabetes with regard to lifestyles, which suggests limited effectiveness of lifestyle interventions in these patients. However, the relationships between aware and unaware diabetes patients were cross-sectional, not permitting drawing causal conclusions, but only suggesting it.

The prevalence of diabetes in our study (6.9%) was lower than in one recent investigation in Spain [29]. Our study participants were younger (50% under 45 years vs. 42%), with a lower proportion of women (50.5% vs. 57.1%) and a lower frequency of obesity in diabetic patients (48% vs. 55%), which could contribute to the observed differences in results. In the US, the prevalence of diabetes (8.6%) [5] is higher than in Spain, which may partly be due to the higher frequency of obesity among aware diabetic patients (62.4%) [5].

One strength of this study is that it provides nationally representative data. However, various study limitations deserve to be considered. First, we excluded 6.7% of study participants because of missing data, and performed a complete-case analysis. Such relatively small levels of exclusion would probably not substantially affect the overall results. Second, we cannot rule out a certain selection bias resulting from using phone interviews. However, it should be noted that over 85% of households in Spain have a fixed phone line. Moreover, a study in Spain has shown that phone interviews had good validity and reproducibility with respect to face-to-face interviews [30]. Third, the sample size was limited for some analyses such as the specific method recommended to stop smoking, which did not allow to compare persons with aware and unaware diabetes. Fourth, the information on medical advice and its adherence was self-reported, therefore it is subject to recall bias and social desirability bias (i.e., providing a socially acceptable answer rather than the most accurate answer). Fifth, we did not distinguish between type 1 and 2 diabetes but it is likely that, as in many developed countries, the majority of patients suffered from type 2 diabetes. Lastly, no information was obtained on pneumococcal immunization.

In conclusion**,** this study in a European country with universal healthcare coverage shows that comprehensive achievement of the cardiometabolic goals and lifestyles recommended by major international guidelines is not good enough. It suggests that improvements in both guideline implementation and patients’ adherence are needed [31].
